# Jumonji AT-rich interactive domain 1B promotes the growth of pancreatic tumors via the phosphatase and tensin homolog/protein kinase B signaling pathway

**DOI:** 10.3892/ol.2023.13660

**Published:** 2023-01-03

**Authors:** Xudong Shen, Guilian Cheng, Liming Xu, Wei Wu, Zhengrong Chen, Peng Du

Oncol Lett 16: 267–275, 2018; DOI: 10.3892/ol.2018.8618

Following the publication of this paper, it was drawn to the Editors’ attention by a concerned reader that certain of the western blotting data shown in Fig. 6A were strikingly similar to data that had appeared in different form in another article by different authors in a different research institution. Owing to the fact that the contentious data in the above article had already been published elsewhere prior to its submission to *Oncology Letters*, the Editor has decided that this paper should be retracted from the Journal. The authors were asked for an explanation to account for these concerns, but the Editorial Office did not receive a reply. The Editor apologizes to the readership for any inconvenience caused.

